# Evaluation of multidimensional pediatric-psychosomatic inpatient therapy: a pilot study comparing two treatment modalities

**DOI:** 10.3389/fpsyg.2023.1022409

**Published:** 2023-06-06

**Authors:** Tim Botschek, Maximilian Monninger, Dennis Schäfer, Rabia Cevik, Kübra Memis, Ulrike Müller, Martina Monninger, Burkhard Brosig

**Affiliations:** ^1^Family Psychosomatics, Department of Pediatrics and Neonatology, Justus-Liebig-University Giessen, Giessen, Germany; ^2^Department of General Pediatrics, University Children’s Hospital Muenster, Münster, Germany; ^3^Sigmund-Freud-Institut, Frankfurt, Germany

**Keywords:** pediatric psychosomatics, multidimensional inpatient therapy, family therapy, alexithymia, treatment duration, pilot study

## Abstract

**Introduction:**

Multidimensional pediatric-psychosomatic inpatient treatment should be considered a highly relevant concept in the German healthcare system. This treatment concept has been successfully integrated to support youth with mental disorders and patients with chronic somatic conditions. Studies on treatment impact and empirical evidence of pediatric-psychosomatic inpatient therapies are rare, despite their clinical significance. Therefore, the study aims to provide initial indications of what constitutes to enhanced treatment effectiveness by comparing two different pediatric-psychosomatic inpatient treatment concepts. The clinics are comparable regarding the treated disorders, which include: dissociative, mood, and somatoform disorders, and psychological factors associated with chronic somatic conditions. Multidimensional treatment in both clinics include components of individual and family therapy, along with group-, art-, music-, creative-, and physio-therapy. Both clinics differed regarding their treatment philosophy in which; Clinic A practiced psychodynamic behavioral elements more strongly, while Clinic B rooted itself more strongly with psychoanalysis and family-dynamic practices.

**Method:**

Each clinic recruited 25 patients for the study. They completed two questionnaires both at admission and discharge, which measured general behavioral and emotional problems (YSR); and, respectively, difficulties in emotion perception and processing (TAS-26). The effectiveness of the treatment was examined by conducting one-sample *t*-test and effect sizes for each clinic. To obtain information on differentiating treatment effects, mixed ANOVAs were calculated. For estimating its influence, the treatment duration was taken into account as a covariate calculating an ANCOVA.

**Results:**

In both settings, treatment effects can be observed regarding internalizing problems. For alexithymia, no effects were seen in Clinic B, while in Clinic A, there was a significant reduction. When comparing both clinics, the ANOVAs showed significant interaction effects displaying advantages for Clinic A in the reduction of internalizing, total behavioral problems and alexithymia. Taking into account the treatment duration as a covariate, those effects level out. Significant differences between the clinics were no longer statistically detectable.

**Discussion:**

The present study provides substantial preliminary indications on the effectiveness of multidimensional pediatric-psychosomatic inpatient therapy, which seems suitable for alleviating the general symptom burden and problems by identifying and processing emotions. Furthermore, the results indicate that an extended treatment duration may contribute to more pronounced effects.

## 1. Introduction

Pediatric-psychosomatic clinics can today be seen as a relevant and well-established concept in the German healthcare system. The “Deutsche Gesellschaft für Pädiatrische Psychosomatik (2022)” [German association of pediatric psychosomatics] lists 44 pediatric-psychosomatic treatment facilities in the country^1^. Pediatric psychosomatics is of high relevance in the care of children and adolescents with mental disorders and in particular in the support of patients with chronic somatic conditions. Many chronic diseases can currently be treated well, but ultimately, they are incurable. The condition of “incurability” may cause considerable mental burden and harm. The multidimensional inpatient setting for psychosomatic disorders generally includes a wide range of therapeutic components and uses different methods, such as nonverbal creative, interpretive-psychological, interactional, and motor approaches to alleviate the symptoms. The clinical relevance of the concept contradicts an enormous lack of research in the evaluation of multidimensional pediatric-psychosomatic inpatient therapy. However, assessments of the effects of psychotherapeutic treatments represent an important ingredient of evidence-based quality assurance and the optimization of patient care.

The present pilot study, therefore, aims to compare the results of inpatient treatment in the pediatric-psychosomatic department of two German university hospitals as part of a cross-institutional cooperation. Differential effects in the alleviation of the symptom burden, which could be traced back to the differences in the treatment concept of the clinics presented below, are to be identified in the present research.

To evaluate the impact of treatment, the patient’s alexithymia will be measured. The concept of alexithymia describes difficulties in identifying, describing, and processing emotions (cf. Kupfer et al., 2001). A three-factorial pattern, consisting of deficits in describing and identifying feelings and an externally oriented style of thinking, was also confirmed for children and younger adolescents (Di Trani et al., 2018). Previous studies indicated the high relevance of the concept in the context of youth mental health. Cerutti et al. (2020) demonstrated that higher levels of alexithymia in children and adolescents correlated with greater somatic and depressive symptom burden and that the association between alexithymia and somatic symptoms was mediated by depressive symptomatology. A more recent study, in the context of the COVID-19 pandemic also concluded an association between alexithymia and somatic symptoms, additionally externalizing problems in children (Renzi et al., 2022). Moreover, the authors demonstrated that the parents’ alexithymia score was associated with both somatic complaints and externalizing problems in their children during the pandemic. The crucial role of the parental emotional capacities in helping their children to regulate their emotional states was also reported by Di Trani et al. (2020). In summary, these results indicate that children and adolescents with limited capabilities to identify and describe feelings tend to process stress-inducing situations in a more somatic, physical way and thereby risk internalizing and expressing their inner states through somatic symptoms, which makes the concept of alexithymia especially relevant in the context of pediatric psychosomatics (see also Renzi et al., 2022).

The presence of internalizing problems served as a second indicator of treatment effects. Internalizing problems include depressive or anxious symptoms and are reported particularly frequently by patients with psychosomatic disorders (Guidetti et al., 2016).

Besides differential treatment effects, this study also shed light on the question of an appropriate duration of treatment. When evaluating inpatient psychiatric and psychosomatic treatments, this aspect repeatedly became the scene of content-related, economic, and ideological debates. Preliminary findings in other contexts suggest a more stable and sustainable treatment success associated with a longer treatment process (Orlinksy et al., 1994; Knekt et al., 2015). In pediatric psychosomatics, however, this aspect has not been investigated yet. The present work should therefore examine possible moderating influences of the treatment duration on the treatment effects. Recourse to empirical data can help to design an efficient treatment that is timely and appropriate to the complexity and fierceness of the mental impairment.

In summary, the present pilot study aims to investigate preliminary indications of the effects of two multidimensional inpatient treatment concepts in pediatric-psychosomatics in order to lay the groundwork for the formulation of specific hypotheses for further research. All hypotheses were formulated in advance and the study was approved by the ethical commissions of both medical departments (Gießen 157/12, amendment from July 16th 2018 and WWU Münster Medical School 2018-708-b-S). In this study, three research questions will be examined:

1. The authors assume that problems with the perception and processing of emotions, measured by the Toronto-Alexithymia-Scale-26 (Kupfer et al., 2001), and the general symptom burden, measured by the syndrome as well as the internalizing problem scale of the Youth Self-Report (Döpfner et al., 2014) can be reduced by means of multidimensional pediatric-psychosomatic inpatient therapy, which would highlight the importance of this concept in clinical care.
2. Furthermore, the two clinics treatment efficacy are to be compared in terms of clinical implementation, therapeutic approach and philosophy, and, in particular, their institutional history. Based on their advantages in experience and clinical implementation, the authors hypothesize greater effects of treatment in Clinic A, indicated by the aforementioned scales of the TAS-26 and the YSR.
3. In addition, the influence of the treatment duration on the outcome of the treatment should be estimated. In line with existing literature, the authors presume that increased treatment duration leads to greater treatment effects, indicated by a greater reduction of the general symptom burden and alexithymia, as measured by the TAS-26 and the YSR.

## 2. Methods

### 2.1. Characteristics of the clinics

Fundamental to the therapeutic conception of both clinics is the view that chronic diseases in adolescents can only be comprehensively understood and therapeutically addressed by considering biographical, social, psychological, and biological aspects. The core element of the particular multidimensional therapy is, thereby proposed as 50-min individual therapy-sessions that occur twice a week and a one-hour family therapy session at least every 2 weeks. The comparable institutional orientation manifests itself on a formal level through the related coding according to the “Operationen- und Prozedurenschlüssel (OPS),” the official classification by which medical operations get reimbursed in Germany.

A common feature of both clinics is an extensive range of educational and creative therapy units, which are however, designed differently: In Clinic A, a more differentiated, patient-centered approach is chosen which individually adapts and varies the different therapeutic modalities to the symptoms presented in the treatment planning. An additional psychotherapeutic-oriented transdiagnostic group therapy takes place once a week to facilitate social–emotional development. Moreover, socio-therapeutic oriented group activities are offered throughout the week to strengthen personal and social abilities. Over the ongoing decades, a wide range of accompanying therapy elements including music-, art-, dance-, animal-assisted-, and physical- therapy, were stepwise introduced and tailored to support the patients in their individual needs. Clinic B, on the other hand, pursues a holistic group-oriented concept in which the aforementioned therapeutic building blocks are identified and utilized as a whole group (Büttner and Brosig, 2021). Here, multidimensional therapy includes art, music, creative and physiotherapeutic modalities as well as yoga. The daily routines are more strongly structured by educationists in Clinic A, while in Clinic B, the structure of everyday life is assigned primarily to pediatric nurses. In Clinic B, there are four obligated group therapy sessions a week, reflecting the focus on a more pronounced group-oriented concept. Table 1 gives an overview of the differences between the two clinics.

**Table 1:** Differences between the two clinics.

	Clinic A	Clinic B
Institutional history, structure and size	Founded 1958	Founded 2017
	19 beds (14 due to COVID-19)	10 beds (9 due to COVID-19)
	Only licensed psychotherapists and medical doctors	Partially psychotherapists in training, licensed medical psychotherapists, pediatrician
Setting and therapeutic modalities	Structured by educationists	Structured by nurses
Special therapeutic features	i.a. animal assisted therapy	i.a. yoga
Treatment philosophy and concept	Individualized, patient-centered, integrative-eclectic	Treatment group-oriented with group sessions 4/week
	Psychodynamic, embedded in a behavioral-pedagogical concept	Psychoanalytic, family-dynamic frame of understanding
Inclusion of the caregivers	Primarily parents counseling	Primarily family therapy
	Advisory	Conflict-centered

Regarding the therapeutic modalities in the sense of the different therapeutic approaches, both clinics utilize a psychodynamic therapeutic approach, following a pediatric psychosomatic complex therapy with a focus on the needs of the individual patient whilst additionally considering their environment including parents and siblings etc. In Clinic A, the therapeutic orientation is focused on individual therapy, embedded in a pedagogical conceptual framework. The approach here aims in particular at changes on the behavioral level. Clinic B grounds itself on the traditionally profound theories of Horst-Eberhard Richter emphasizing his psychoanalytic and family-dynamic approaches in practice (Richter, 1970, 1972). In Clinic A, the therapeutical inclusion of the caregivers is regarded more strongly in the sense of parents counseling and an advisory exchange, while in Clinic B, family therapy is characterized by the attempt to bring awareness to previously unconscious family conflicts and relational issues in order to intervene accordingly, in a conflict-centered manner.

Both Clinics can be considered long-term oriented, which is reflected in the treatment durations which are generally several months, when necessary. Based on the different institutional history, however, Clinic A is more experienced in performing long-term treatments and thereby are able to cope better with the different dynamics and processes that come along with inpatient long-term treatment, which can be challenging for both the patients and the team members. These issues include occurring difficulties with the long absence from home or intensive, emotionally charged therapeutic processed, which can be very upsetting for the patient.

The two clinics can be described as very restrictive when it comes to pharmacological interventions. Pharmacotherapy is not regarded as a genuine part of the treatment in both clinics.

Besides distinctions in the treatment philosophies, there are also differences in terms of the size and, in particular, the experiences made on the ward and with the team, as well as the institutional history. While Clinic A has a maximum of 19 beds, in Clinic B, there are no more than 10 beds available. Due to the hygiene-related restrictions in the context of the COVID-19 pandemic, a maximum of 14, respectively, 9 beds could be occupied at the time of the survey. In general, Clinic A is a well-established ward of a general pediatric clinic with a long-standing history. Its roots date back to the late 1950s. In contrast, Clinic B has been in existence for 6 years and is therefore a comparatively young institution. In Clinic A, individual therapy sessions are constantly performed by clinical psychological therapists as well as medical doctors trained in different therapeutic methods, including cognitive behavioral therapy, systemic psychotherapy, or depth psychology. In contrast, in Clinic B, therapy sessions are also performed regularly by psychotherapists in training, with little clinical experience. Therapists here are trained predominantly in depth and analytic therapy, unfrequently in systemic and behavioral therapy.

### 2.2. Sample characteristics

Twenty-five patients aged 12 to 18 years (Clinic A: *M* = 14.88, *SD* = 1.64, Clinic B: *M* = 14.24, *SD* = 1.59) were recruited from each clinic. Regarding age, there were no significant differences between the clinics, *t*(48) = −1.401, *p* = 0.168. The gender ratio also showed no significant differences. While seven (28%) male patients were treated in clinic A, this proportion was 36% or nine patients in Clinic B.

The treatment duration ranged between 3 and 26 weeks. The clear divergent duration of treatment has complex origins. On the one hand, different problem constellations require treatments of different intensity over time (Warnke and Rössler, 2008). This seems to be associated with the various degrees of psychological and familial impairments in the symptoms described, so that mental recovery can be achieved in some cases in a briefer time than in the others. Moreover, to maintain the naturalistic character of the study, patients who discontinued the treatment ahead of time against medical advice and therefore only had a short stay in the respective clinics were also included. We thereby assured the inclusion of the whole spectrum of patients treated in both clinics, reflecting the clinical reality of different treatment durations. The two study samples can therefore be considered representative of the clinical samples regularly treated in the context of both clinics.

Forty-four patients from Clinic A and, respectively, 31 patients from Clinic B were treated during the study period. Reasons for the exclusion from the study were language barriers, age, lack of consent to participate in the study, or organizational reasons of the wards, which led to the corresponding test battery not being answered. Following the finalization of 25 complete data sets, the survey was terminated.

The indications for multidimensional psychosomatic treatment in both clinics are comparable. These include affective, dissociative, somatoform, and eating disorders. Furthermore, patients with chronic conditions such as diabetes mellitus or chronic inflammatory bowel diseases receive therapeutic support in terms of mental processing, acceptance, and coping (Meister et al., 2013; Kunert and Meister, 2019). Table 2 summarizes the distribution of the treated disorders.

**Table 2:** Treated disorders coded according to ICD-10.

Diagnoses	Clinic A *N* (Percentage)	Clinic B *N* (Percentage)
Mood (Affective) disorder (F3)	3 (12)	3 (12)
Neurotic, stress-related and somatoform disorders (F4)	18 (72)	13 (52)
Behavioral syndromes associated with physiological disturbances and physical factors (F5)	1 (4)	8 (32)
Disorders of personality and behavior (F6)	0 (0)	1 (4)
Behavioral and emotional disorders with onset usually occurring in childhood and adolescence (F9)	3 (12)	0 (0)

From the treatment spectrum presented, basic comparability of the treated disorders can be assumed, although slightly different emphases can be stated. In both clinics, disorders from the domain of neurotic and somatoform disorders and the reaction to severe stress, coded under F4 in the ICD-10 (World Health Organization, 1992), represent the largest treatment group. This applies in particular to Clinic A. In Clinic B patients with adaptation and coping problems associated with physiological disturbances (ICD-10: F5) are treated more frequently. Patients with acute psychoses, substance use disorders, delinquency, or acute suicidality are excluded from treatment and thus also from the present study in both clinics.

### 2.3. Instruments

The patients recruited from both clinics filled out two questionnaires each on admission (T_1_) and discharge (T_2_), the Youth Self Report (YSR; Döpfner et al., 2014) and the Toronto-Alexithymia-Scale-26 (TAS-26; Kupfer et al., 2001).

The concept of alexithymia dates back to the work of John Case Nemiah and Peter Emanuel Sifneos in the 1970s (Nemiah and Sifneos, 1970). This term describes the difficulty individuals have in identifying, processing, and describing feelings, which is particularly common in patients with psychosomatic disorders (Kupfer et al., 2001). Alexithymia can be assessed using the Toronto-Alexithymia-Scale (TAS), which was developed by Taylor and Bagby in 1994 (Bagby et al., 1994a,b) and transferred into German by Kupfer, Brosig, and Brähler. This version, not the one with 20 items (TAS-20), was used, given that it is better established in the German-speaking world, mainly because there is a German text manual available. The TAS-26 is also superior to the TAS-20 referring to its psychometric quality characteristics. It is composed of three subscales, which measure difficulties in identifying feelings (Scale 1), difficulties in describing feelings (Scale 2), and externally oriented thinking (Scale 3). The aforementioned scales can then be summed up to a total alexithymia score. The German version of the TAS-26 demonstrated satisfying internal consistency scores (Cronbach’s alpha between 0.67 and 0.84 for the subscales and 0.81 for the total scale).

The YSR assesses general behavioral and emotional problems. A total problem score (“syndrome scale”), as well as scores for externalizing and internalizing problems, can be interpreted. Regarding psychosomatic complaints, internalizing problems in particular were reported in previous studies (Guidetti et al., 2016). The YSR demonstrates high internal consistency scores (Cronbach’s Alpha 0.82 for the internalizing problem scale, 0.93 for the total problem scale).

Of particular interest in the context of psychosomatic treatment and therefore the subject of the analysis are the scales of the internalizing and overall problems of the YSR as well as the total alexithymia scale and the three subscales of the TAS-26.

### 2.4. Statistical analyses

For the statistical analysis, one-sample *t*-tests were first conducted and the effect sizes were calculated to determine the outcome of the treatment in both settings. To obtain information on possible differential effects in the treatment, 2 (Clinic A/B) × 2 (T_1_/T_2_) mixed ANOVAs were then calculated. The interaction effect, which describes the alteration in the particular outcome variables over time depending on the respective clinic, was of primary interest. In order to be able to make statements about the influence of the treatment duration on the results found, repeated measures ANCOVAs, in which the treatment duration was taken into account as a covariate, were finally calculated.

## 3. Results

Evaluation of the patients’ alexithymia, using the TAS-26, yielded significant effects in reducing difficulties in identifying [Scale 1; *t*(49) = 3.156, *p* = 0.003, *d* = 0.49] and describing feelings [Scale 2; *t*(49) = 2.201, *p* = 0.033, *d* = 0.33] by the inpatient treatment when considering both clinics combined (see Table 3). No effects were found for the externally oriented thinking, *t*(49) = 0.350, *p* = 0.728, *d* = 0.06. The total alexithymia score was significantly lower at discharge compared to the time of admission when evaluating both clinics combined, *t*(49) = 2.94, *p* = 0.005, *d* = 0.28. Looking at both clinics individually, some relevant differences emerge. While alexithymia, estimated using the TAS-26 total scale, is significantly reduced in Clinic A [*t*(24) = 3.914, *p* = 0.001, *d* = 0.78], there is no effect in Clinic B [*t*(24) = 0.339, *p* = 0.738, *d* = 0.07]. A more differentiated picture emerges by evaluating the subscales. In Clinic A, both the difficulties in identifying and describing feelings are reduced statistically significantly [Scale 1: *t*(24) = 2.563, *p* = 0.017, *d* = 0.51, Scale 2: *t*(24) = 2.221, *p* = 0.036, *d* = 0.44], while in Clinic B there were no significant differences between the time of discharge and admission for the scales mentioned [Scale 1: *t*(24) = 1.942, *p* = 0.064, *d* = 0.39, Scale 2: *t*(24) = 0.746, *p* = 0.463, *d* = 0.15]. Regarding the externally oriented thinking, there were no differences between admission and discharge at either clinic [Clinic A: *t*(24) = 0.974, *p* = 0.340, *d* = 0.19, Clinic B: *t*(24) = −1.598, *p* 0.123, *d* = 0.32]. Considering the mean values, an even slightly more externally oriented style of thinking can be observed in Clinic B upon discharge.

**Table 3:** Psychometric effects of pediatric-psychosomatic inpatient therapy in Clinic A and B.

Instrument	Setting	*M (SD)* T_1_	*M (SD)* T_2_	*t*-value	Sig.	Effect size (Cohen’s *d*)
YSR						
Syndrome scale						
	Clinic A	60.48 (24.36)	39.64 (19.31)	5.676	<0.001**	1.14
	Clinic B	52.76 (28.63)	43.92 (29.63)	1.916	0.067	0.38
	Total (A and B)	56.62 (26.60)	41.78 (24.85)	4.880	<0.001**	0.58
Internalizing problems						
	Clinic A	24.44 (12.03)	13.40 (9.59)	5.458	<0.001**	1.09
	Clinic B	19.56 (12.04)	15.16 (12.83)	2.121	0.044*	0.42
	Total (A and B)	22.00 (12.16)	14.28 (11.25)	5.112	<0.001**	0.66
TAS						
Total						
	Clinic A	54.44 (10.47)	48.40 (9.64)	3.914	0.001**	0.78
	Clinic B	46.84 (12.38)	46.36 (12.92)	0.339	0.738	0.07
	Total (A and B)	50.64 (11.98)	47.38 (11.33)	2.94	0.005**	0.28
Scale 1						
	Clinic A	21.32 (9.66)	16.00 (5.67)	2.563	0.017*	0.51
	Clinic B	16.60 (6.97)	14.40 (7.14)	1.942	0.064	0.39
	Total (A and B)	18.96 (8.67)	15.20 (6.43)	3.156	0.003**	0.49
Scale 2						
	Clinic A	16.88 (5.97)	14.08 (4.52)	2.221	0.036*	0.44
	Clinic B	13.64 (4.72)	12.92 (5.48)	0.746	0.463	0.15
	Total (A and B)	15.26 (5.57)	13.50 (5.01)	2.201	0.033*	0.33
Scale 3						
	Clinic A	21.00 (14.64)	18.32 (3.40)	0.974	0.340	0.19
	Clinic B	17.40 (4.99)	19.04 (6.27)	−1.598	0.123	0.32
	Total (A and B)	19.20 (10.98)	18.68 (5.00)	0.350	0.728	0.06

YSR, Youth Self Report; TAS, Toronto-Alexithymia-Scale-26; Scale 1, Difficulties in identifying feelings; Scale 2, Difficulties in describing feelings; Scale 3, Externally oriented thinking. *p < 0.05, **p < 0.01.

The YSR paints a slightly different picture. Analyzing the general symptom burden, significant effects can be reported when looking at both clinics combined. For both the internalizing problem scale [*t*(49) = 5.112, *p* < 0.001, *d* = 0.66] and the total problem scale [*t*(49) = 4.880, *p* < 0.001, *d* = 0.58], a significant reduction in symptoms was found. Looking at the two clinics individually, treatment effects can be observed concerning internalizing problems, which in the course of treatment both in Clinic A [*t*(24) = 5.458, *p* < 0.001, *d* = 1.09] and in Clinic B [*t*(24) = 2.121, *p* = 0.044, *d* = 0.42] are statistically significantly reduced. With regard to the more comprehensive syndrome scale, a significant reduction in the symptom burden can be stated for Clinic A [*t*(24) = 5.676, *p* < 0.001, *d* = 1.14], while the change in Clinic B does not reach statistical significance [*t*(24) = 1.916, *p* = 0.067, *d* = 0.38], although the effect size found indicates a small effect (Cohen, 1988).

To evaluate different treatment effects depending on the clinic, the Mixed Model ANOVAs yielded the following results (see Table 4). Regarding the YSR, both the syndrome scale [*F*(1,48) = 4.142, *p* = 0.047] and the scale of internalizing problems [*F*(1,48) = 5.252, *p* = 0.026] revealed a significant Clinic x time point interaction effect. Therefore, the change in the overall problems and the internalizing problems during the inpatient treatment varied depending on the Clinic visited, with Clinic A proven to be superior in this respect.

**Table 4:** Differentiate treatment effects and influence of the treatment duration, examined with mixed method ANOVAs and ANCOVAs.

Instrument	Stat. analysis	*F*-value	Sig.
YSR			
Syndrom scale			
	ANOVA	4.142	0.047*
	ANCOVA	2.177	0.147
Internalizing problems			
	ANOVA	5.252	0.026*
	ANCOVA	3.008	0.089
TAS			
Total			
	ANOVA	7.044	0.011*
	ANCOVA	3.114	0.084
Scale 1			
	ANOVA	1.741	0.193
	ANCOVA	0.261	0.612
Scale 2			
	ANOVA	1.716	1.96
	ANCOVA	0.356	0.553
Scale 3			
	ANOVA	2.163	1.48
	ANCOVA	0.792	0.378

YSR, Youth Self Report; TAS, Toronto-Alexithymia-Scale-26; Scale 1, Difficulties in identifying feelings; Scale 2, Difficulties in describing feelings; Scale 3, Externally oriented thinking. *p < 0.05.

A significant interaction effect with advantages for Clinic A can also be reported for the total scale of the TAS-26 [*F*(1,48) = 7.044, *p* = 0.011]. The extent of the change in alexithymia over treatment, therefore, varies significantly depending on the clinic. However, no significant interaction effects can be observed for the three subscales.

When estimating the influence of the duration of treatment, it should first be noted that the length of hospitalization is significantly longer in Clinic A than in Clinic B [Clinic A: *M* = 14.6, *SD* = 6.72, Clinic B: *M* = 10.0, *SD* = 5.24; *t*(48) = 2.70; *p* = 0.010].

If the duration of treatment is now taken into account as a covariate executing an ANCOVA, the previously significant interaction effect levels out. Significant differences in the reduction of both behavioral and emotional problems in the syndrome scale [*F*(1,47) = 2.177, *p* = 0.147], and the scale of internalizing problems in the YSR [*F*(1,47) = 3.008, *p* = 0.089], depending on the treatment setting, are *no longer statistically detectable*. The same applies to the total scale of the TAS-26 [*F*(1,47) = 3.114, *p* = 0.084].

To draw more precise conclusions about the influence of the treatment duration on the effects of the inpatient treatment, these results were analyzed using a stepwise multiple regression on the scales of internalizing problems; total symptom burden; and the total alexithymia scale at the time of discharge, considering the treatment duration, the clinic and the respective baseline values as predictors.

It was shown for the total scale of the TAS-26, that including the treatment duration as a predictor improves the prognostic validity statistically significantly. The baseline value of alexithymia (*ß* = 0.805, *p* < 0.001), and the duration of treatment (*ß* = −0.345, *p* = 0.049), contribute significantly to the prediction of the total alexithymia value at discharge. The goodness of fit of the model is high (*R^2^* = 0.632), and reached statistical significance [*F*(2,47) = 40.303, *p* < 0.001].

Regarding the YSR syndrome scale, in contrast, it can be stated that only the baseline value contributes significantly to predicting the symptom burden at discharge (*ß* = 0.638, *p* < 0.001). The treatment duration leads to a non-significant improvement in the goodness of fit of the model (*ß* = −0.347, *p* = 0.447). The same pattern applies to the internalizing problems scale.

## 4. Discussion

Firstly, the present work aimed to examine whether multidimensional pediatric-psychosomatic inpatient therapy is suitable for significantly reducing the burden of symptoms and difficulties in the perception and processing of emotions in children and adolescents with psychosomatic disorders. Secondly, the treatment outcomes of two German clinics with slightly different treatment concepts were compared and examined for divergent effects. Thirdly, the authors were interested in the influence of the treatment duration on therapeutic outcomes.

The reported results are meaningful clinically and in the context of health policy. On the one hand, it can be indicated impressively that in psychosomatic disorders in childhood and adolescence, a multidimensional pediatric-psychosomatic inpatient therapy appears to have significant effects in the diminution of symptoms, alteration of perceptions and the ability to process emotions. This becomes especially evident when analyzing the results of both clinics combined. These findings agree with previous studies in which the efficacy of multidimensional pediatric-psychosomatic treatment was reported (Zessin, 2017) and highlight the importance and relevance of such treatment in effectively treating psychosomatic complaints in children and adolescents. Pediatric psychosomatic medicine thereby fills an otherwise deplorable gap in the care of patients who have problems accepting and coping with their disease, but who have no other option of inpatient, e.g., psychiatric, treatment because of their existing somatic diseases. Interdisciplinary collaboration also allows a careful differential diagnostic, which is of great importance given the growing number of initially unclear somatic complaints, which also increased during the COVID-19 pandemic (Ravens-Sieberer et al., 2013). The often complex complaints, which include somatic, psychological, and psychosocial problems, family conflicts, school absenteeism, and other problems, seem to require an equally complex medical-therapeutic approach, which can often only be provided within the framework of multidimensional inpatient therapy. The results reported here underline the effectiveness of such a treatment for the problems described.

Concerning the question of differential treatment effects depending on the respective clinic, it should first be emphasized that for both clinics significant results in terms of the diminution of symptoms can be reported. However, Clinic A has an advantage over Clinic B in terms of both reducing symptom burden and overcoming alexithymia. In addition to the different treatment durations discussed below, there are various causes to consider. In the beginning, reference was made to the differences between the two clinics. Clinic A, with its numerous years of experience, can be seen as a much more established institution whose structures and institutional networks are more recessed. In addition, Clinic A mostly works with already licensed child and adolescent therapists, while Clinic B also employs numerous therapists in training in addition to medical specialists in psychosomatic medicine and psychotherapy. References to the importance of clinical experience for therapeutic success can be found elsewhere (Tschuschke et al., 2015), although the results are contradictory (Germer et al., 2022).

Furthermore, when assessing the different treatment results, the somewhat diverse therapeutic approach of the two clinics must be considered. In other studies, especially in adults, behavioral therapy (BT) has proven itself to be superior to other, e.g., psychoanalytic, methods when it comes to the question of rapid symptom diminution. In the long term, the results of BT often approximate those of other methods (Leichsenring et al., 2014; Zipfel et al., 2014). Such a development has to be examined in more detail in the future, for example through catamnestic examinations.

When interpreting the divergent treatment effects, the difference in the diagnoses treated should be taken into consideration. As Table 2 shows, the group of neurotic, stress-related, and somatoform disorders (ICD-10: F4) are treated most frequently in both clinics. Nevertheless, there are some relevant differences in the distribution of the presented disorders. While the percentage of the aforementioned neurotic, stress-related, and somatoform disorders is higher in Clinic A, in Clinic B there were more cases of treatment of adjustment and coping problems associated with somatic diseases (ICD-10: F5). This group of disorders compromises on the one hand psychological aspects of compliance and coping with physical problems and disorders, but also eating disorders, especially anorexia nervosa (ICD-10: F50.0). This clinical picture has proven itself to be especially reluctant and complex when it comes to psychotherapeutic interventions (Abbate-Daga et al., 2013). The notably higher proportion of patients with this type of disorder in Clinic B compared to Clinic A could therefore be part of the explanation for the slightly differential treatment outcomes.

In the present study, the duration of treatment proved to be significant in explaining the divergent treatment effects. The reported results indicate that the previously found differences between the two clinics level out with increasing treatment duration. This finding is in line with other previous studies that could relate greater treatment success to longer treatment duration (Orlinksy et al., 1994; Knekt et al., 2015). This seems to apply in particular to fundamental aspects of psychopathology such as alexithymia as a marker of emotion processing. This finding is consistent with the basic consideration that aspects of character or personality are subject to slow changes, while intensive therapeutic support can reduce the burden of symptoms more efficiently.

There was a wide range of treatment durations included in the present study, which can be considered representative of the clinical reality of treating psychosomatic disorders in adolescents and, thus, highlights the naturalistic character of the study. On the other hand, it reflects the major differences between the two clinics when it comes to their different institutional history. As described above, Clinic A is a long-standing, well-established psychosomatic clinic and an essential part of the general pediatrics at its location. It is considerably more experienced in dealing with extensive treatment durations and their special challenges like intense and upsetting treatment processes. In contrast, Clinic B has only been established for a few years and has disadvantages in terms of clinical expertise and inclusion in institutional structures. Extraordinary long durations of treatment are rare, although over the course of the last years, the consolidation of clinical structures have permitted increasingly longer durations of treatment in Clinic B. The different durations of treatment therefore reflect the subtle, yet meaningful institutional differences in terms of history and clinical experience and are therefore a relevant and significant indicator when comparing both clinics.

In summary, in order to bolster therapeutic effects, e.g., reducing symptoms, achieve lasting effects and initiating a process toward the normalization of pathological character traits most effectively, a longer treatment period seems indicated. This should also be considered when approving accordant treatments.

Limitations of the study relate to the sample characteristics, especially the rather small sample size, which reflects the character of a pilot study. Replication of the effects in larger samples is therefore indicated. For that manner, a power analysis should be executed *a priori* to allow an accurate appraisal of the participants needed. For future research, information on previous hospitalization or additional pharmacotherapies should be considered as additional covariates of interest in order to allow a more detailed comparison between both samples. We did not integrate pharmacotherapy as a variable of interest in the statistical analysis due to its rare occurrence in both clinics. Furthermore, both treatment approaches include elements of family therapy or counseling, thereby emphasizing the crucial role of the caregivers in the therapeutic process. Future research should therefore take that issue into account by including questionnaires filled out by the parents. Furthermore, catamnestic surveys can be informative in future investigations in order to allow statements about the sustainability of the effects achieved.

The comparison of the two clinics is also problematic because the therapies involved are not manualized. The differences between the treatments were summed up above, but the analysis allows no more precise conclusions about which factors, apart from the duration of treatment, could be the cause of the diverse effects found. In future research, other factors of interest such as the experience of the therapists etc., could be included as covariates. Nevertheless, the comparison of the two clinics appears reasonable given the similar treatment standard according to the OPS, the comparable treatment indication, and the contrastable clinical concepts.

In addition, the present research lacks an untreated control group to separate randomly occurring remission effects from therapeutically induced effects. However, it must be taken into account that in the case of acute inpatient treatment in the context of pediatric psychosomatics, it is required that outpatient pretreatments were unsuccessful. In addition, the acuteness of symptoms generally does not ethically allow for a waiting period due to the potential risk of harm inflicted from postponing necessary treatment, as is the case in the waiting-control group designs.

In summary, the present pilot study provides substantial preliminary indications of the effectiveness of a multidimensional pediatric-psychosomatic inpatient therapy, which seems suitable for alleviating the problems of patients with numerous existing psychosomatic symptoms. The shortcomings may provide adjusted categories for further evaluations of pediatric psychosomatics. Further research in this field is indicated and hopefully stimulated by the reported findings to verify these results using larger samples, additional relevant variables and a long-term catamnesis.

## Data availability statement

The raw data supporting the conclusions of this article will be made available by the authors, without undue reservation.

## Ethics statement

The studies involving human participants were reviewed and approved by the Ethik-Kommission am Fachbereich Medizin, Justus-Liebig-Universität Gießen. Written informed consent to participate in this study was provided by the participants’ legal guardian/next of kin.

## Author contributions

TB, RC, KM, MarM, and BB contributed to conception and design of the study. TB performed the statistical analysis. TB, DS, and BB wrote the first draft of the manuscript. All authors accompanied and monitored the clinical implementation of the study and contributed to manuscript revision, read, and approved the submitted version.

## Conflict of interest

The authors declare that the research was conducted in the absence of any commercial or financial relationships that could be construed as a potential conflict of interest.

The reviewer FL declared a shared affiliation with the authors TB, DS, KM, UM, and BB to the handling editor at the time of review.

## Publisher’s note

All claims expressed in this article are solely those of the authors and do not necessarily represent those of their affiliated organizations, or those of the publisher, the editors and the reviewers. Any product that may be evaluated in this article, or claim that may be made by its manufacturer, is not guaranteed or endorsed by the publisher.

## Footnotes

^1^ https://www.agpps.de/index.php/einrichtungen
